# The Surgery of the Tongue

**Published:** 1892-10

**Authors:** 


ARTICLE VI.

### THE SURGERY OF THE TONGUE.

In an excellent paper on this subject read before the American Surgical Association, May 31, 1892, Dr. Dandridge presented the following conclusions:

1. Sufficient experience has been accumulated to show that removal of cancer of the tongue prolongs life, adds to the comfort of the patient, and further affords a reasonable hope of permanent cure.

2. All operations should be preceded by an effort to secure thorough disinfection of the mouth and teeth.

3. In the treatment of continued ulcers and sores on the tongue, caustics are to be avoided and all sources of irritation removed.

4. Persistent sores on the tongue should be freely removed by knife or scissors if they resist treatment.

5. When the disease is confined to the tongue, Whitehead's operation should be employed for its removal.

6. In this operation the advantage of preliminary ligation of the lingual artery is not definitely settled, but the weight of authority is against its necessity.

7. The advantage of leaving one-half the tongue in unilateral disease must be considered undetermined, but the weight of positive experience is in its favor. In splitting the tongue into lateral halves, Baker's method of tearing through the raphe should always be employed.

8. A preliminary tracheotomy adds an unnecessary element of danger in the removal of the tongue in ordinary cases.

9. When the floor of the mouth has become involved, or the glands are enlarged, Kocher's operation should be employed, omitting the spray and preliminary tracheotomy.

10. Removal of the glands by a separate incision after the removal of the tongue, must be considered insufficent.

11. Volkmann's method still rests on individual experience. Its just value cannot be determined until it has been subjected to trial by a number of surgeons.

12. Thorough and complete removal should be the aim of all operations, whether for limited or extreme disease.

13. By whatever method the tongue is removed, the patient should be up and out of bed at the earliest possible moment, and should be generously fed.—*Journal of the American Medical Association.*

